# Silencing XIST mitigated lipopolysaccharide (LPS)-induced inflammatory injury in human lung fibroblast WI-38 cells through modulating miR-30b-5p/CCL16 axis and TLR4/NF-κB signaling pathway

**DOI:** 10.1515/biol-2021-0005

**Published:** 2021-02-06

**Authors:** Jiahui Xu, Honggui Li, Ying Lv, Chang Zhang, Yiting Chen, Dezhao Yu

**Affiliations:** Department of Pediatrics, Guangdong Second Hospital of Traditional Chinese Medicine, No. 60 Hengfu Road, 510000, Guangzhou, Guangdong, China; Department of Pediatrics, The First Affiliated Hospital of Guangzhou University of Traditional Chinese Medicine, 510000, Guangzhou, Guangdong, China; Department of Pediatrics, The Fifth Clinical College of Guangzhou University of Traditional Chinese Medicine, 510000, Guangzhou, Guangdong, China; Department of Internal Medicine, Guangdong Second Hospital of Traditional Chinese Medicine, 510000, Guangzhou, Guangdong, China; Department of Pediatrics, The Affiliated Zhujiang Hospital of Southern Medical University, 510000, Guangzhou, Guangdong, China

**Keywords:** XIST, miR-30b-5p, LPS-induced WI-38 cells, CCL16, pneumonia, TLR4/NF-κB

## Abstract

**Background:**

Emerging evidence shows that long noncoding RNA (lncRNA) has been a novel insight in various diseases, including pneumonia. Even though lncRNA X-inactive-specific transcript (XIST) is well studied, its role in pneumonia remains to be largely unrevealed.

**Methods:**

Expression of XIST, miRNA-30b-5p (miR-30b-5p), and CC chemokine ligand 16 (CCL16) was detected using reverse transcriptase quantitative polymerase chain reaction and western blotting; their interaction was confirmed by dual-luciferase reporter assay. Apoptosis, inflammation, and toll-like receptor 4 (TLR4)/NF-κB signaling pathway were measured using methyl thiazolyl tetrazolium assay, flow cytometry, western blotting, and enzyme-linked immunosorbent assay.

**Results:**

Lipopolysaccharide (LPS) stimulation decreased cell viability and B cell lymphoma (Bcl)-2 expression, and increased cell apoptosis rate and expression of Bcl-2-associated X protein (Bax), cleaved-caspase-3, interleukin (IL)-6, IL-1β, and tumor necrosis factor α (TNF-α) in WI-38 cells. Expression of XIST and CCL16 was upregulated in the serum of patients with pneumonia and LPS-induced WI-38 cells, respectively; silencing XIST and CCL16 could suppress LPS-induced apoptosis and inflammation in WI-38 cells, and this protection was abolished by miR-30b-5p downregulation. Moreover, XIST and CCL16 could physically bind to miR-30b-5p, and XIST regulated CCL16 expression via sponging miR-30b-5p. TLR4 and phosphorylated P65 (p-P65) and p-IκB-α were highly induced by LPS treatment, and this upregulation was diminished by blocking XIST, accompanied with CCL16 downregulation and miR-30b-5p upregulation.

**Conclusions:**

Silencing XIST could alleviate LPS-induced inflammatory injury in human lung fibroblast WI-38 cells through modulating miR-30b-5p/CCL16 axis and inhibiting TLR4/NF-κB signaling pathway.

## 1 Introduction

Pneumonia is a leading infectious cause of death, especially in the young and old people [1,2]. Infantile pneumonia and community-acquired pneumonia are common types of pneumonia. The infection caused by pathogenic microorganisms, such as bacteria, mycoplasma, and viruses, leads to typical clinical symptoms of pneumonia including fever, cough with purulent sputum or blood, chest pain, and shortness of breath [3]. Naturally, pneumonia is an inflammatory disease in lower respiratory tracts [4]. Lipopolysaccharide (LPS) is the main bioactive component of Gram-negative bacteria pathogens [5] and can induce severe inflammatory response in lungs [6,7]. Therefore, LPS-induced inflammatory injury is a popular model for studying the pathogenesis and management of pneumonia.

Long non-coding RNA (lncRNA), with over 200 nucleotides, is a group of transcripts with little protein coding capacity. Emerging evidence suggests that lncRNA has been a novel insight in various diseases, such as lung injuries [8]. In pneumonia, a potential key lncRNA profile in the peripheral blood has been identified using lncRNA sequencing [9]. Several lncRNAs have been further disclosed to be associated with inflammatory response in lungs [10,11,12]. LncRNA X-inactive-specific transcript (XIST) is one of the best studied lncRNAs [13]. Previous studies show a link between XIST and inflammation [14,15]. Unfortunately, to date, the role of XIST in the inflammatory response in pneumonia remains largely unknown.

It is well known that lncRNA can act as a competitive endogenous RNA (ceRNA) to modulate microRNA (miRNA) activity, thus affecting the pathological and physiological processions, and so does XIST [15,16]. MiRNA (miR)-30b-5p has been reported to participate in inflammatory injury in several tissues, including kidney, cartilage, and lung [17,18,19]. Thus, we put forward a hypothesis that there might be an interaction between XIST and miR-30b-5p in pneumonia.

In this study, we’ve examined expression of XIST and miR-30b-5p in the serum of patients with pneumonia. The role of both in inflammatory injury in cell model of pneumonia was confirmed in LPS-induced human fibroblast WI-38 cells *in vitro*. Furthermore, the molecular mechanism of XIST in regulating LPS-induced apoptosis and inflammation was evaluated, revealing that it’s acting as a ceRNA and regulating a well known LPS-related signaling pathway, toll-like receptor 4 (TLR4)/NF-κB which is well known as an [20].

## 2 Materials and methods

### 2.1 Collection of serum samples

The peripheral venous blood (3 mL) was collected from 30 acute stage patients with pneumonia (20 males and 10 females; average age, 20.3 ± 4.0 years) and 30 healthy volunteers (20 males and 10 females; average age, 21.7 ± 3.5 years) in Guangdong Second Hospital of Traditional Chinese Medicine. The diagnosis of pneumonia was confirmed according to the diagnostic criteria of acute pediatric pneumonia, and the clinical features of patients with pneumonia are presented in Table 1. Patients with other complications or who received anti-inflammatory treatment before were excluded. The control blood samples were from people with normal physical examination results. After collection, the blood was centrifuged at 2,000 rpm, and then the supernatant was obtained as serum samples and stored at −80°C.

**Table 1:** Clinicopathological features of patients with acute pneumonia

Clinicopathological features (total = 30)		
Gender	Female	10
	Male	20
Age	≧20	16
	≦20	14
LDH (U/L)	345–386	21
	302–344	9
White blood cell count (×10^9^/L)	8.0–8.6	20
	7.5–8.0	10
Absolute neutrophils (×10^9^/L)	1.9–2.3	22
	1.3–1.9	8

LDH, lactate dehydrogenase.


**Informed consent:** Informed consent was obtained from all individuals included in this study.
**Ethical approval:** The research related to human use has been complied with all the relevant national regulations, institutional policies, and in accordance with the tenets of the Helsinki Declaration and has been approved by the Ethics Committee of Guangdong Second Hospital of Traditional Chinese Medicine.

### 2.2 Cell culture and cell transfection

The human lung fibroblast cell line WI-38 (American Type Culture Collection; CCL-75) was cultured in ATCC-formulated Eagle’s Minimum Essential Medium containing 10% fetal bovine serum (Invitrogen, Carlsbad, CA, USA). The cells in logarithmic phase were transfected with nucleotides using Lipofectamine RNAiMAX (Invitrogen) according to the manufacturer’s protocol. Oligonucleotides including short hairpin RNAs against XIST and CCL16 (sh-XIST/CCL16), miR-30b-5p mimic and inhibitor, as well as their negative controls were purchased from GenePharma (Shanghai, China) and were transfected with a final concentration of 50 nM; plasmids pcDNA3.1 expressing XIST or not were transfected with 2 μg. For co-transfection, half nucleotides were used. After transfection for 24 h, WI-38 cells were harvested for further detections.

### 2.3 LPS-induced cell model of pneumonia

Commercial LPS (L4391) was purchased from Sigma-Aldrich (St. Louis, MO, USA) and was dissolved in dimethyl sulfoxide (DMSO; Sigma-Aldrich) at a stock concentration of 1 mg/mL. WI-38 cells and transfected WI-38 cells in 80% confluence were treated with LPS (5, 10, and 20 μg/mL) for 24 h or 10 μg/mL LPS for 0–48 h. The control group was treated with 0.1% DMSO, and all groups contained not more than 0.1% of DMSO to avoid obvious toxicity.

### 2.4 Total RNA and protein isolation

WI-38 cells under LPS treatment for 24 h were washed twice using cold phosphate buffer solution (PBS). Then, the total RNA in the serum samples and WI-38 cells was lysed in Trizol reagent (Invitrogen), and the protein was extracted by radioimmunoprecipitation assay (Invitrogen). All the procedures were performed according to the manufacturer’s instructions. After isolation, the concentrations of RNA and protein were determined by NanoDrop 2000 (Thermo Scientific, Wilmington, DE, USA) and BCA™ Protein Assay Kit (Pierce, Appleton, WI, USA), respectively.

### 2.5 Reverse transcriptase quantitative polymerase chain reaction (RT-qPCR)

Expression of RNAs was evaluated by RT-qPCR. About 500 ng of total RNA was used to synthesize cDNA using iScript cDNA Synthesis Kit (Bio-Rad, Hercules, CA, USA), followed by amplification of cDNA with iQ SYBR Green Supermix (Bio-Rad) on CFX96 Real-Time System (Bio-Rad). The reactions were performed in triplicate for each sample, and glyceraldehyde phosphate dehydrogenase (GAPDH) and U6 small nuclear RNA (U6) were used as internal controls to XIST, CCL16, and miR-30b-5p. The primers of these genes were listed as follows: XIST, 5′-CGGGTCTCTTCAAGGACATTTAGCC-3′ (forward) and 5′-GCACCAATACAGAGGAATGGAGGG-3′ (reverse); CC chemokine ligand 16 (CCL16), 5′-GCCCACTGAGAGGATGAAGG-3′ (forward) and 5′-TACTTCAGGCAGCAGTTGGG-3′ (reverse); miR-30b-5p, 5′-GGCGTGTAAACATCCTACACTC-3′ (forward) and 5′-GTGCAGGGTCCGAGGT-3′ (reverse); GAPDH, 5′-CTCTGCTCCTCCTGTTCGAC-3′ (forward) and 5′-ACCAAATCCGTTGACTCCGA-3′ (reverse); and U6, 5′-CTCGCTTCGGCAGCACA-3′ (forward) and 5′-AACGCTTCACGAATTTGCGT-3′ (reverse). The relative expression of genes was calculated depending on the threshold cycle (Ct) using the 2^−∆∆Ct^ method, and then normalized to controls.

### 2.6 Cell viability assay

Cell viability of WI-38 cells was measured with methyl thiazolyl tetrazolium (MTT; Sigma Aldrich) staining. LPS-treated cells at 24 h were incubated with 20 μL of MTT (5 mg/mL in FBS-free medium) for 4 h. Then, the formazan crystals was dissolved with 100 μL of DMSO (Sigma Aldrich). The optimal density (OD) at 490 nm was read on Benchmark Plus™ microplate spectrometer (Bio-Rad). The result of each group was presented as the average of three independent treatments and normalized to control.

### 2.7 Flow cytometry

Cell apoptosis was examined with Annexin V-fluorescein isothiocyanate (FITC) and propidium iodide (PI) apoptosis detection kit (BD Biosciences, Franklin Lakes, NJ, USA). After LPS stimulation for 24 h, WI-38 cells were harvested and washed with cold PBS. The cell pellet was resuspended in Annexin V-binding buffer, followed by incubation with 10 µL mixture of Annexin V-FITC and PI in dark at 37°C for 15 min. Finally, 400 µL of binding buffer was added in the cells, and the cells were analyzed on CellQuest software (BD Biosciences) with a FACSCalibur flow cytometer (BD Biosciences). Apoptosis rate was the percentage of cells in Annexin V+/PI and Annexin V+/PI+ quadrants.

### 2.8 Enzyme-linked immunosorbent assay (ELISA)

After LPS stimulation for 24 h, the product of pro-inflammatory cytokines in the supernatant culture was determined by ELISA. Interleukin (IL)-6 kit (ab208113, 1:10,000), IL-1β kit (ab2105; 1:1,000), and tumor necrosis factor α (TNF-α) kit (ab6671, 1:1,000) were obtained from Abcam. The experiment was performed three times for each group and the absorbance at 450 nm was read using Benchmark Plus™ microplate spectrometer (Bio-Rad).

### 2.9 Western blotting

Expression of proteins was examined by western blotting. The standard procedures including SDS-polyacrylamide gel electrophoresis, membrane transference, antibodies incubation, and enhanced chemiluminescence detection were applied for 20 μg of total protein. The primary antibodies including CCL16 (ab199162, 1:2,000), B cell lymphoma (Bcl)-2 (ab196495, 1:2,000), Bcl-2-associated X protein (Bax; ab199677, 1:1,000), cleaved-caspase-3 (ab49822, 1:500), IL-6 (ab208113, 1:1,000), IL-1β (ab2105; 1:1,000), TNF-α (ab6671, 1:2,000), and GAPDH (ab8245, 1:10,000) were provided by Abcam (Cambridge, UK). Primary antibodies against TLR4 (sc-293072, 1:500) and phosphorylated P65 (p-P65; sc-136548, 1:500) and p-IκB-α (sc-8404, 1:500) were obtained from Santa Cruz (Shanghai, China). The polyvinylidene fluoride (Millipore, Billerica, MA, USA) membrane used for protein transfer was blocked with 5% non-fat milk at room temperature for 1 h. Then, the membranes were incubated with the aforementioned primary antibodies at 4°C overnight and reincubated with horseradish peroxidase-labeled secondary antibodies against Rabbit IgG (ab205718, 1:50,000; Abcam) and Mouse IgG (ab97023, 1:20,000; Abcam) at room temperature for 2 h. The relative protein levels were normalized to GAPDH and then compared to control.

### 2.10 Dual-luciferase reporter assay

Starbase (http://starbase.sysu.edu.cn) was used to predict the target gene of XIST and miR-30b-5p, and the dual-luciferase reporter assay was used to confirm the findings. The wild type (WT) of XIST and 3′-untranslated region of CCL16 (CCL16 3′-UTR) were cloned into psi-CHECK-2 (Promega, Madison, WI, USA), as well as the mutant type (MUT). WI-38 cells were co-transfected with WT/MUT-XIST and miR-30b-5p/NC mimic, or co-transfected with CCL16 3′-UTR-WT/MUT and miR-30b-5p/NC mimic. After 24 h, 100 µL of luciferase assay reagent II and 100 µL of 1× Stop&Glo^®^ reagent (Promega) were added to the cells. Finally, the *Firefly* and *Renilla* luciferase activities were read on GloMax^®^ Discover Multimode Microplate Reader (Promega). The data were presented as relative luciferase activity normalized to *Renilla* and then compared to control. This was repeated three times for every group.

### 2.11 Statistical analysis

All data are expressed as mean ± standard deviation from three independent experiments in Tables A1–A8. Statistical analysis was performed using one-way analysis of variance on Graphpad Prism 5 (GraphPad, San Diego, CA, USA). *P*-value less than 0.05 was considered to be statistically significant.

## 3 Results

### 3.1 XIST was upregulated and miR-30b-5p was downregulated in the serum of patients with pneumonia and LPS-induced cell model of pneumonia

**Figure 1:**
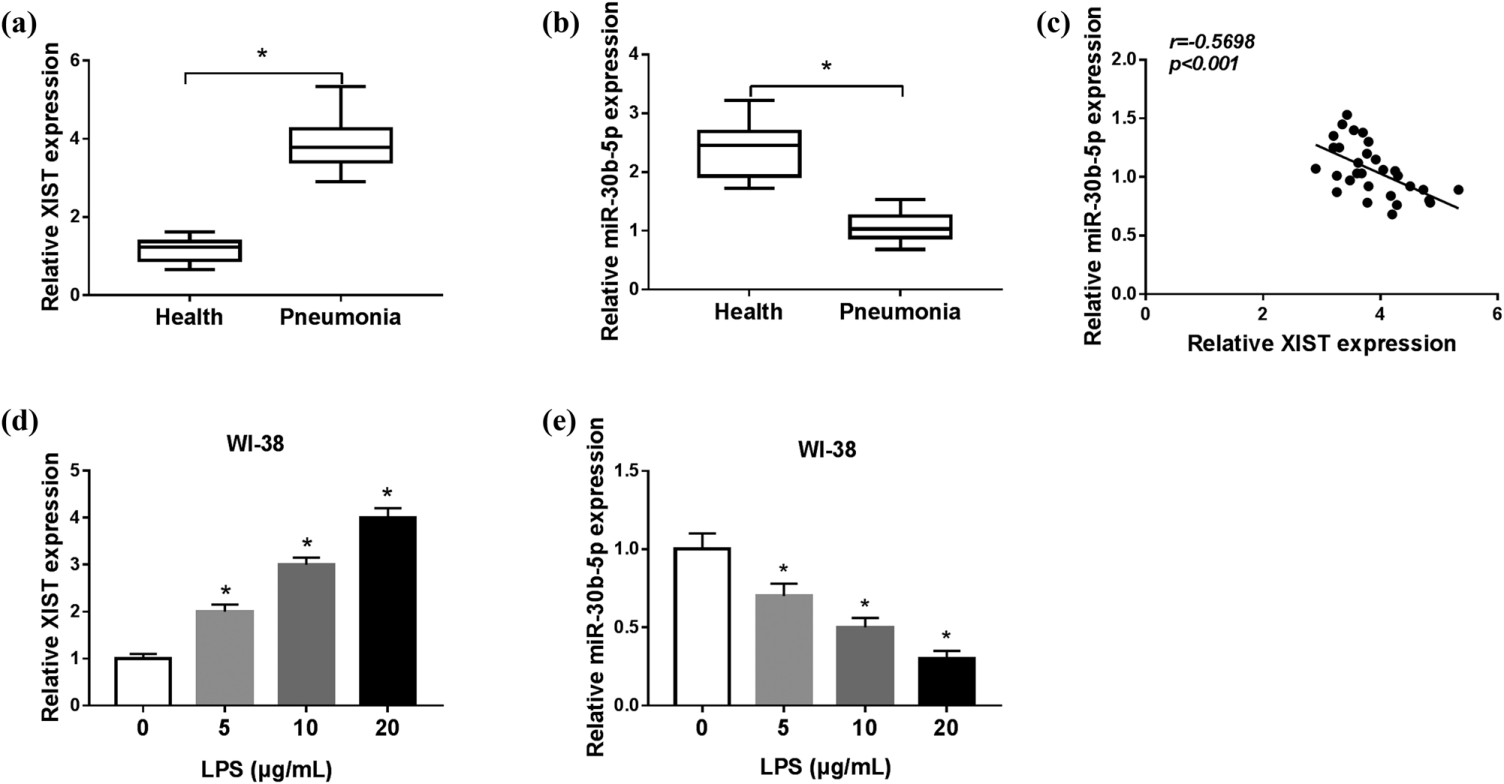
Expression of lncRNA XIST (XIST) and miRNA (miR)-30b-5p in patients with pneumonia and LPS-induced human lung fibroblast cells. RT-qPCR detected XIST and miR-30b-5p levels in (a and b) the serum of patients with pneumonia (*n* = 30) and healthy controls (*n* = 30), and in (d and e) WI-38 cells under LPS treatment (0, 5, 10, and 20 μg/mL) for 24 h. (c) Spearman’s rank correlation analysis testified the correlation between XIST and miR-30b-5p expression in patients with pneumonia. *P < 0.05.

**Figure 2:**
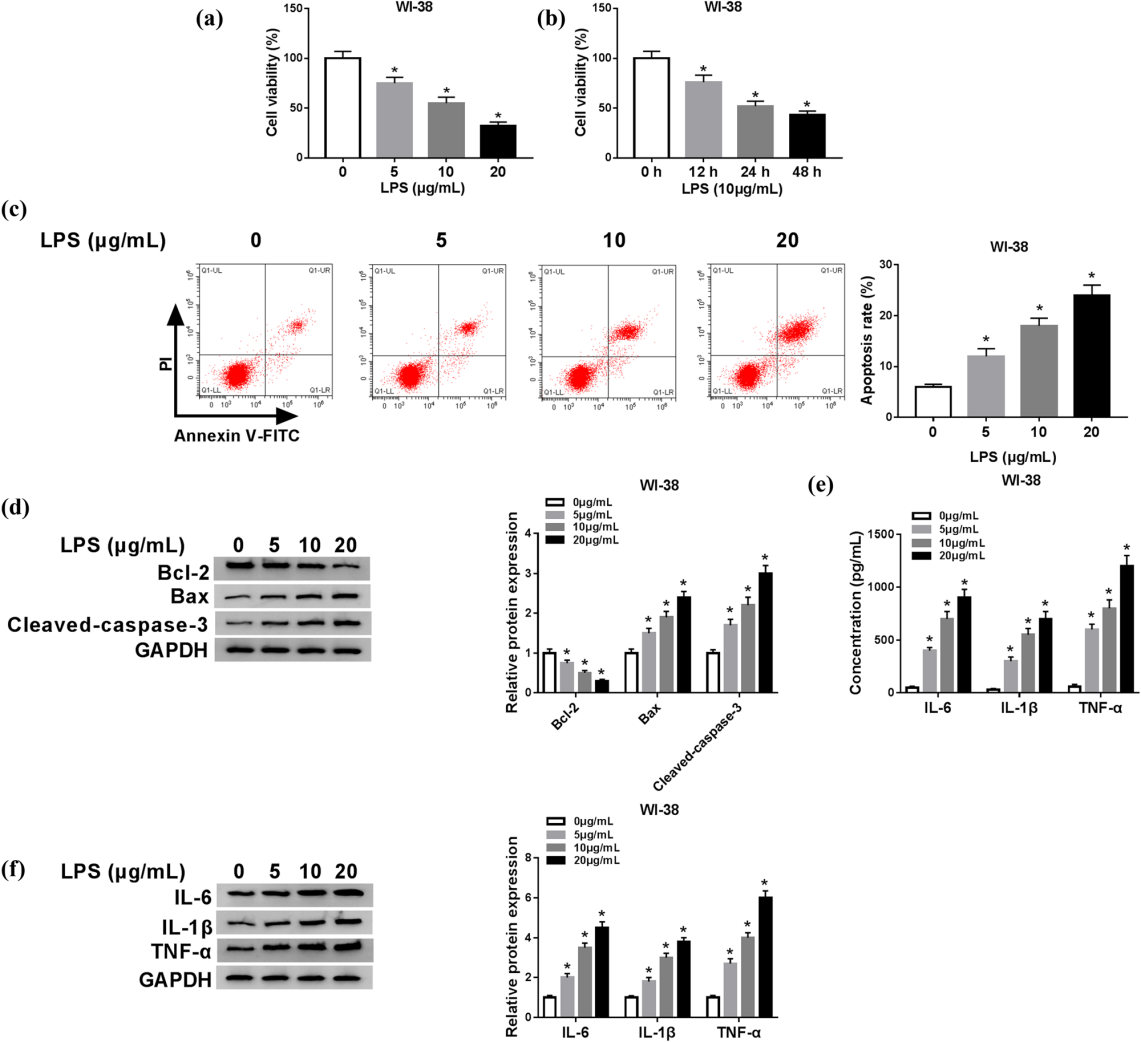
LPS induced apoptosis and inflammation in human lung fibroblast cells *in vitro*. WI-38 cells were treated with 0, 5, 10, and 20 μg/mL of LPS for 24 h or 10 μg/mL of LPS for 0–48 h. (a and b) Cell viability was measured by MTT assay. (c) Apoptosis was evaluated by flow cytometry and apoptosis rate was calculated. (d) Expression of Bcl-2, Bax, and cleaved caspase-3 was measured by western blotting. (e) Expression of IL-6, IL-1β, and TNF-α in cells and (f) secretion of IL-6, IL-1β, and TNF-α in culture supernatant were determined by western blotting and ELISA, respectively. *P < 0.05.

We examined a total of 60 serum samples including 30 patients with pneumonia and 30 healthy controls. As shown in Figure 1a and b, the expression level of XIST was abundantly increased, but that of miR-30b-5p was significantly decreased in the serum from patients with pneumonia compared to the controls. Moreover, there was an inverse correlation between XIST and miR-30b-5p expression in patients with pneumonia (Spearman’s rank correlation analysis, *r* = 0.5698, *P* < 0.001; Figure 1c). This outcome suggested a potential role of XIST and miR-30b-5p in pathogenesis of pneumonia, as well as a potential interplay between both genes. LPS was used to treat human lung fibroblast cells for establishment of *in vitro* model of pneumonia. The dose of LPS treatment was determined in WI-38 cells. After LPS stimulation (5, 10, and 20 μg/mL) for 24 h, XIST level was elevated, whereas miR-30b-5p was attenuated with the growing concentration of LPS (Figure 1d and e). Then, the inflammatory was confirmed in WI-38 cells under LPS treatment, as well. MTT assay showed that cell viability was distinctively weakened with 5, 10, and 20 μg/mL of LPS treatment for 24 h (Figure 2a), and with 10 μg/mL of LPS treatment for 12–48 h (Figure 2b). Flow cytometry and western blotting data indicated that cell apoptosis was dramatically induced in WI-38 cells under 5, 10, and 20 μg/mL of LPS treatment for 24 h, as shown by higher apoptosis rate and expression of Bax and cleaved-caspase-3, and lower Bcl-2 level (Figure 2c and d). In terms of pro-inflammatory factors, IL-6, IL-1β, and TNF-α were highly expressed in WI-38 cells with a significant secretion into the culture medium, as shown by ELISA and western blotting assays (Figure 2e and f). Moreover, 10 μg/mL of LPS treatment for 24 h was good enough (about 50% cell viability inhibition) to induce WI-38 cells (Figure 2b). These outcomes showed that LPS could induce cell apoptosis and inflammation in WI-38 cells, and XIST and miR-30b-5p were abnormally expressed in patients with pneumonia and LPS-treated WI-38 cells.

### 3.2 Knockdown of XIST alleviated LPS-induced apoptosis and inflammation in human lung fibroblast WI-38 cells

**Figure 3:**
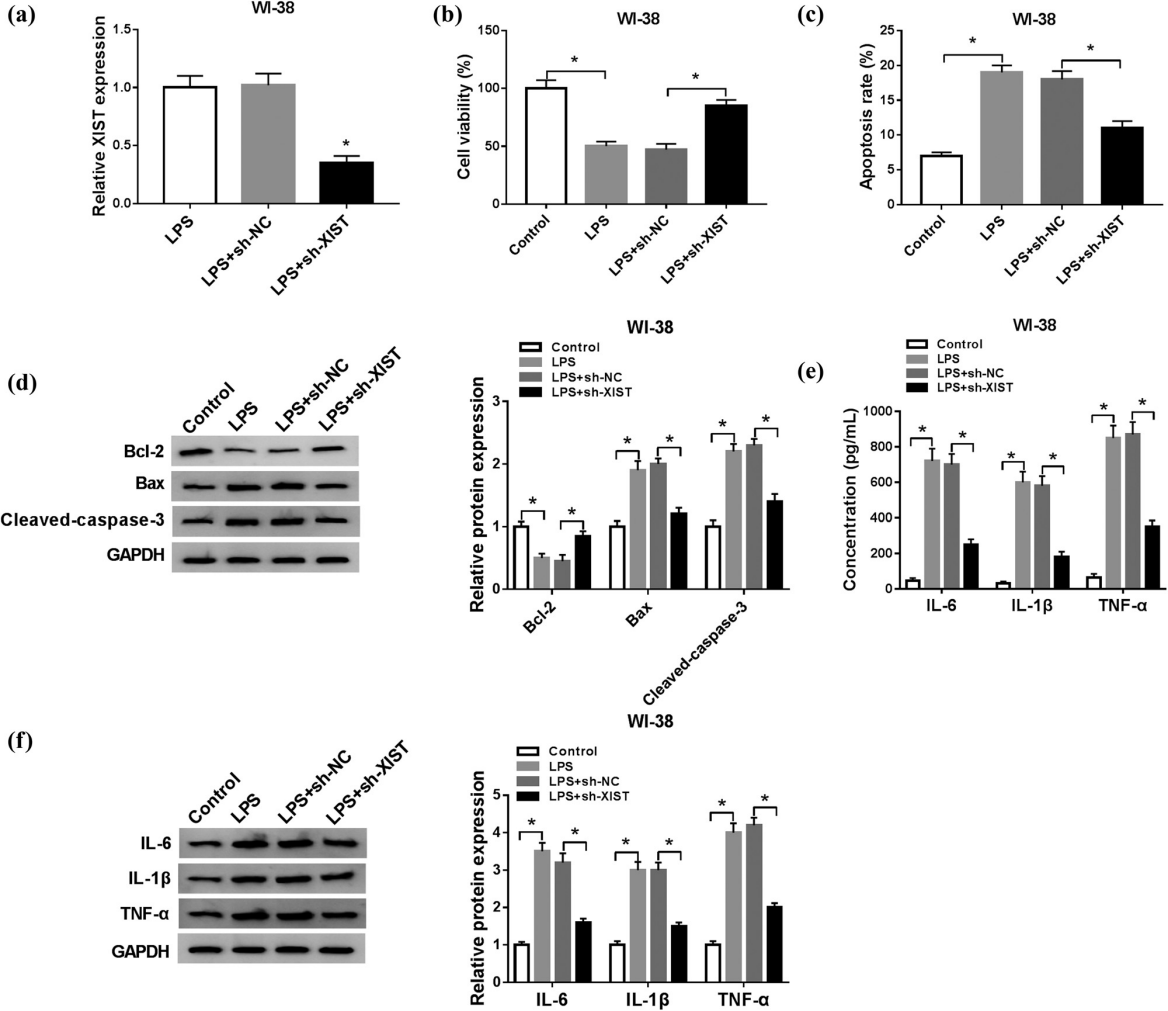
Knockdown of XIST alleviated LPS-induced apoptosis and inflammation in human lung fibroblast cells *in vitro*. WI-38 cells were transfected with sh-XIST or sh-NC, and then treated with 10 μg/mL of LPS for 24 h. After LPS stimulation, (a) RT-qPCR determined the levels of XIST, (b) MTT assay measured cell viability, (c) flow cytometry evaluated cell apoptosis, (d) western blotting measured Bcl-2, Bax, and cleaved caspase-3 levels, and (e and f) ELISA and western blotting examined levels of IL-6, IL-1β, and TNF-α in cells and culture supernatant. *P < 0.05.

To explore the effect of XIST in cell model of pneumonia in human lung fibroblast cells, WI-38 cells transfected with sh-XIST or sh-NC were treated with 10 μg/mL of LPS. The results in Figure 3a show that sh-XIST transfection could significantly decrease XIST expression in LPS-induced WI-38 cells. The cell viability inhibition in LPS-induced WI-38 cells at 24 h was reversed by XIST knockdown (Figure 3b); on the contrary, the promotion of LPS on apoptosis rate at 24 h, as well as expression of Bax and cleaved-caspase-3, was abated when XIST was forcedly lower expressed (Figure 3c and d). LPS treatment induced high expression of IL-6, IL-1β, and TNF-α in WI-38 cells and secreted in the supernatant culture, and this effect was diminished by sh-XIST transfection (Figure 3e and f). These results suggested a protective role of XIST knockdown in LPS-induced cell model of pneumonia in human lung fibroblast cells by reducing cell apoptosis and inflammation.

### 3.3 Protective role of XIST knockdown depended on upregulating miR-30b-5p through sponging

**Figure 4:**
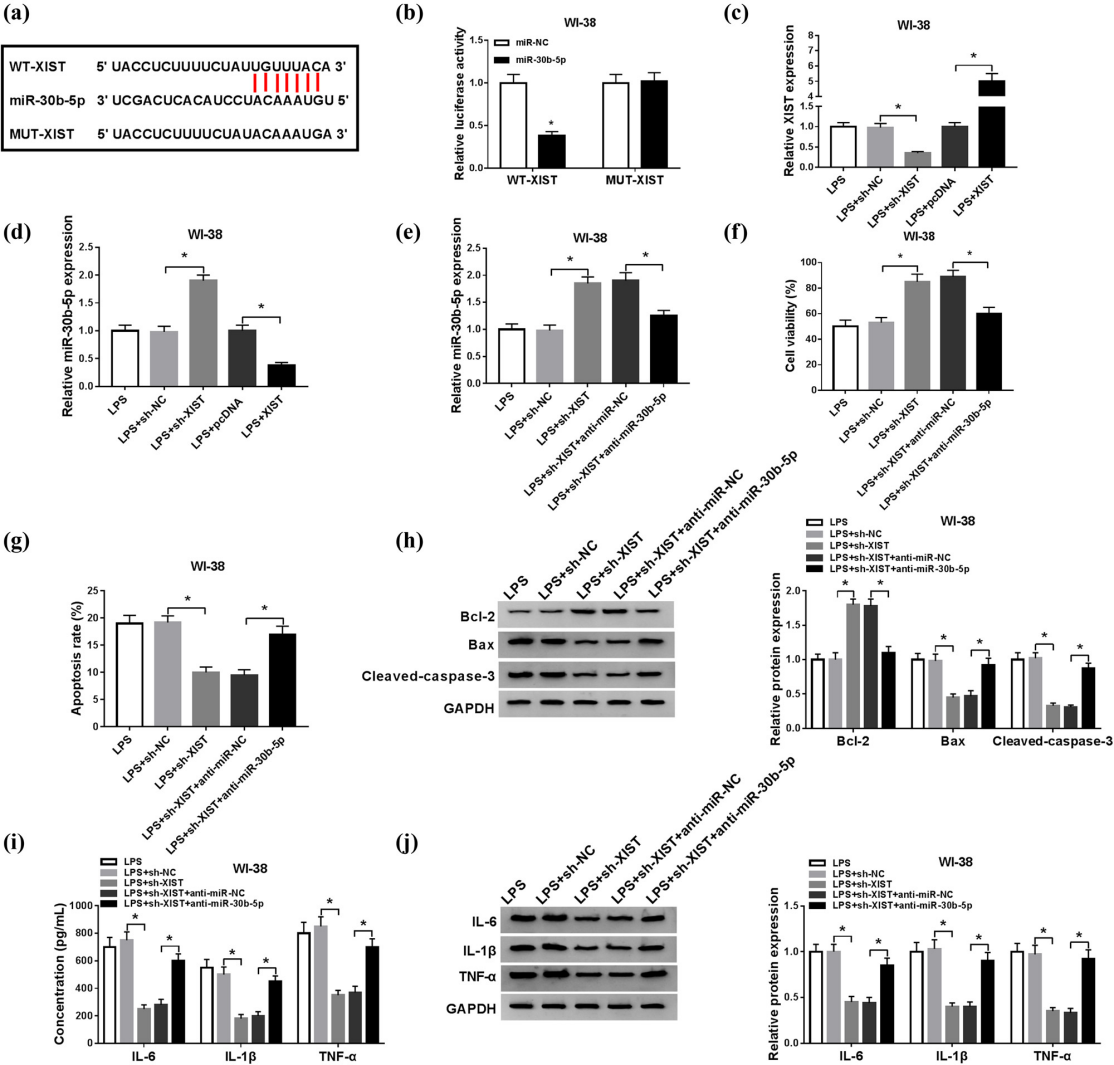
The protective role of XIST knockdown depended on upregulating miR-30b-5p through sponging. (a) The putative binding site between XIST and miR-30b-5p was presented. (b) The relative luciferase activities of WT and mutant of XIST (WT/MUT-XIST) were examined by dual-luciferase reporter assay. (c and d) RT-qPCR detected levels of XIST and miR-30B-5p in WI-38 cells which were transfected with sh-XIST, XIST overexpressing vector (XIST), or the negative controls, and then exposed to 10 μg/mL of LPS. (e–j) WI-38 cells were transfected with sh-XIST or sh-NC, and co-transfected with sh-XIST and anti-miR-30b-5p or anti-NC, followed by treatment of 10 μg/mL of LPS for 24 h. After LPS stimulation, (e) RT-qPCR determined the levels of XIST at 24 h, (f) MTT assay measured cell viability, (g) flow cytometry evaluated cell apoptosis, (h) western blotting measured Bcl-2, Bax, and cleaved caspase-3 levels, and (i and j) ELISA and western blotting examined levels of IL-6, IL-1β, and TNF-α in cells and culture supernatant. *P < 0.05.

Based on these results, we wondered whether there was a direct interaction between XIST and miR-30b-5p in human lung fibroblast cells. The target binding site between both was searched in starBase v2.0 database (http://starbase2/lncRNA XIST-hsa-mir-30b-5p). *In silico* data showed a conserved complementary paired site of miR-30b-5p in XIST (Figure 4a). To further analyze this target binding, the putative target sequence UGUUUAC in the WT of XIST was mutated into the complementary sequence. Dual-luciferase reporter assay was performed to evaluate the relative luciferase activities of vectors containing WT-XIST and MUT-XIST. As shown in Figure 4b, only the WT-XIST group possessed significantly lower luciferase activity when transfected with miR-30b-5p mimic. Moreover, the regulatory effect of XIST on miR-30b-5p was also evaluated. RT-qPCR analysis showed that, with a higher transfection efficiency of sh-XIST and pcDNA-XIST (Figure 4c), the expression level of miR-30b-5p in WI-38 cells was upregulated when transfected with sh-XIST and downregulated when transfected with pcDNA-XIST (Figure 4d). Subsequently, a series of rescue experiments were launched in WI-38 cells transfected with sh-XIST or sh-NC, and co-transfected with sh-XIST and anti-miR-30b-5p or anti-NC, followed by treatment of 10 μg/mL of LPS. First, the increase in miR-30b-5p level mediated by sh-XIST was partially eliminated with anti-miR-30b-5p introduction (Figure 4e). The inducing effect of XIST knockdown on cell viability in LPS-treated WI-38 cells at 24 h was attenuated when miR-30b-5p was deleted by transfection (Figure 4f). On the contrary, the inhibitory effects of XIST knockdown on apoptosis rate and expression of Bax and cleaved-caspase-3 in LPS-treated WI-38 cells at 24 h were partially improved with anti-miR-30b-5p transfection (Figure 4g and h), as well as on product of IL-6, IL-1β, and TNF-α in cells and culture supernatant (Figure 4i and j). Taken together, XIST knockdown exerted a protective role in LPS-induced apoptosis and inflammation, at least, through sponging miR-30b-5p.

### 3.4 MiR-30b-5p negatively regulated CCL16 expression in human lung fibroblast WI-38 cells

**Figure 5:**
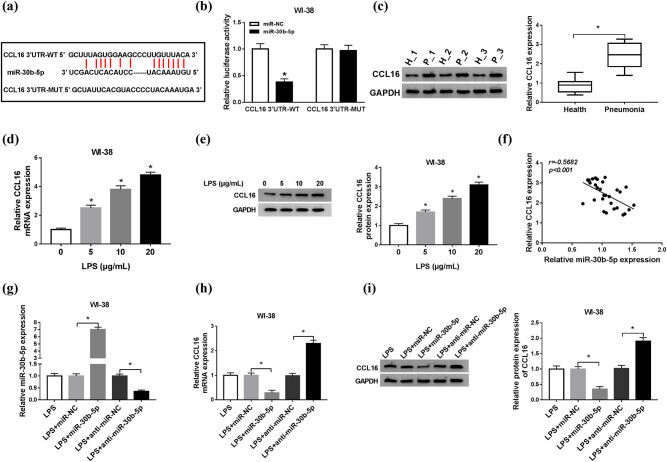
miR-30b-5p negatively regulated CCL16 expression in human lung fibroblast cells *in vitro*. (a) The putative binding site between miR-30b-5p and CCL16 was presented. (b) The relative luciferase activities of WT and mutant of CCL16 3′-UTR (CCL16 3′-UTR-WT/MUT) were examined by dual-luciferase reporter assay. (c) RT-qPCR evaluated mRNA expression level of CCL16 in the serum of patients with pneumonia (*n* = 30) and healthy controls (*n* = 30). Three serum samples in both groups were selected to confirm CCL16 protein expression levels by western blotting. (d and e) Levels of CCL16 in LPS-induced WI-38 cells were measured by RT-qPCR and western blotting. (f) Spearman’s rank correlation analysis testified the correlation between miR-30b-5p and CCL16 mRNA expression in patients with pneumonia. (g–i) RT-qPCR and western blotting measured miR-30b-5p and CCL16 levels in WI-38 cells which were transfected with miR-30b-5p mimic (miR-30b), anti-miR-30b-5p, or the negative controls, and then treated with 10 μg/mL of LPS for 24 h. *P < 0.05.

The functional target gene of miR-30b-5p was further identified. According to starBase v2.0 database (http://starbase/mirnas&target=CCL16), there was a potential binding sequence of miR-30b-5p in the 3′-UTR of CCL16. As shown in Figure 5a, the putative sites in the WT of CCL16 3′-UTR were mutated to the complementary sites. After co-transfection with miR-30b-5p mimic, vectors containing CCL16 3′-UTR-WT showed a significant decline in the relative luciferase activity than co-transfection with miR-NC mimic (Figure 5b). Then, the expression status of CCL16 in pneumonia was established. Data from RT-qPCR and western blotting depicted a considerably higher level of CCL16 in the serum from patients with pneumonia (*n* = 30; Figure 5c). Besides, its expression was increased stepwise in WI-38 cells with different doses of LPS (Figure 5d and e). Moreover, there has been a negative correlation between CCL16 and miR-30b-5p expression in patients with pneumonia (Spearman’s rank correlation analysis, *r* = 0.5682, *P* < 0.001; Figure 5f). The regulatory effect of miR-30b-5p on CCL16 was also evaluated. RT-qPCR analysis showed a higher transfection efficiency of miR-30b-5p mimic and anti-miR-30b-5p in LPS-induced WI-38 cells (Figure 5g), and expression level of CCL16 was upregulated when transfected with anti-miR-30b-5p and downregulated when transfected with miR-30b-5p mimic (Figure 5h and i). These results indicated that CCL16 was a direct target of miR-30b-5p in human lung fibroblast cells.

### 3.5 Silencing of CCL16 migrated LPS-induced apoptosis and inflammation in human lung fibroblast WI-38 cells relying on miR-30b-5p upregulation

**Figure 6:**
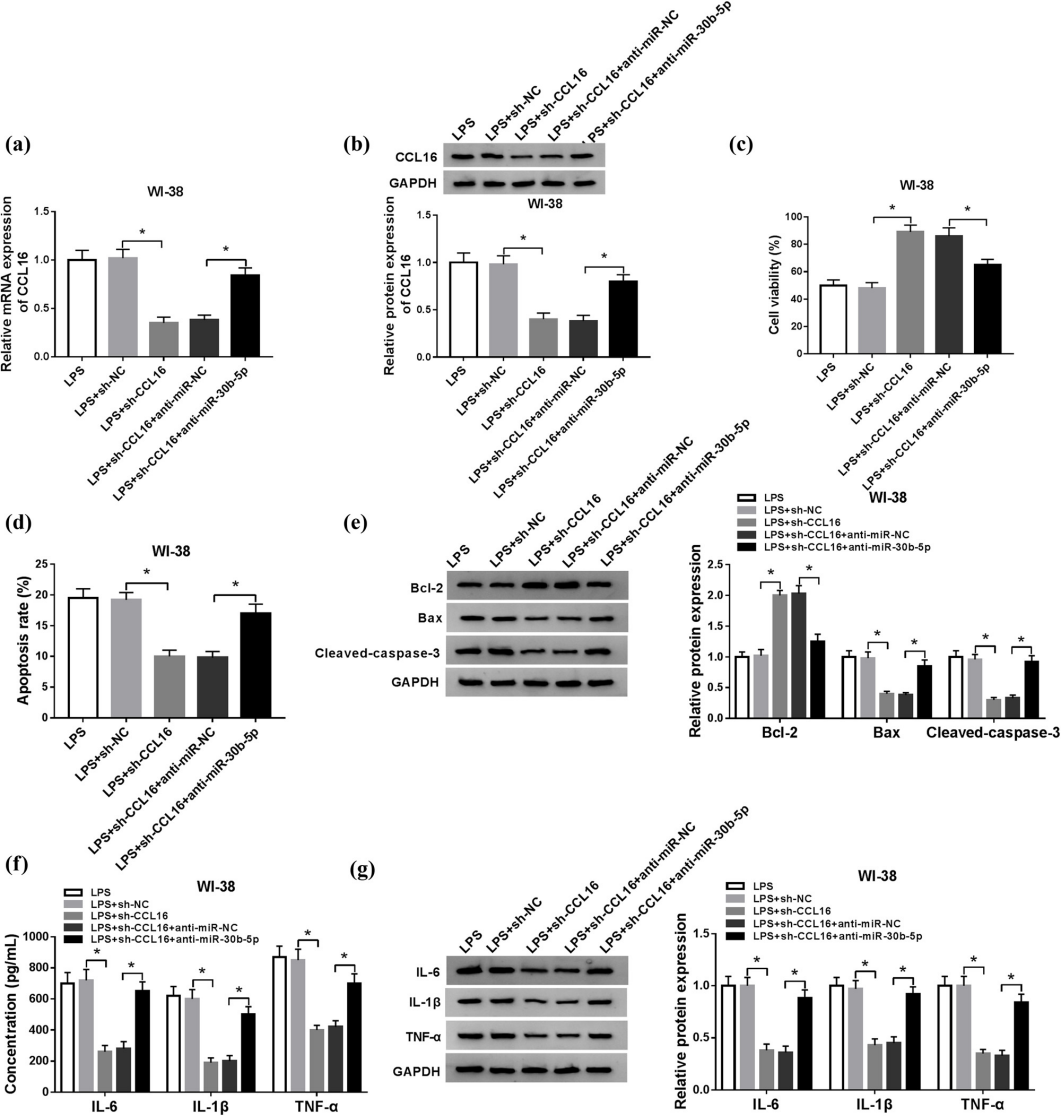
Silencing of CCL16 migrated LPS-induced apoptosis and inflammation in human lung fibroblast cells *in vitro* relying on miR-30b-5p upregulation. WI-38 cells were transfected with sh-CCL16 or sh-NC, co-transfected with sh-CCL16 and anti-miR-30b-5p or anti-NC, and then treated with 10 μg/mL of LPS for 24 h. After LPS stimulation, (a and b) RT-qPCR and western blotting determined the levels of CCL16, (c) MTT assay measured cell viability, (d) flow cytometry evaluated cell apoptosis, (e) western blotting measured Bcl-2, Bax, and cleaved caspase-3 levels, and (f and g) ELISA and western blotting examined levels of IL-6, IL-1β, and TNF-α in cells and culture supernatant. *P < 0.05.

The role of CCL16 in cell model of pneumonia in human lung fibroblast cells was further detected, as well as the influence of miR-30b-5p expression on this role. WI-38 cells were transfected with sh-CCL16 or sh-NC, and then treated with 10 μg/mL of LPS. Results in Figure 6a and b show that sh-CCL16 transfection could significantly decrease CCL16 mRNA and protein expression in LPS-induced WI-38 cells. Because of CCL16 silencing, the cell viability of LPS-induced WI-38 cells at 24 h was greatly enhanced (Figure 6c), but the apoptosis was dropped at 24 h as evidenced by loss of apoptosis rate and expression of Bax and cleaved-caspase-3, and increase in Bcl-2 level (Figure 6d and e). LPS treatment induced higher expression of IL-6, IL-1β, and TNF-α in WI-38 cells and secreted in the supernatant culture, and this effect was diminished with sh-CCL16 transfection (Figure 6f and g). These outcomes suggested a suppressive role of CCL16 downregulation in LPS-induced inflammatory injury in human lung fibroblast cells *in vitro*. Moreover, when co-transfected with sh-CCL16 and anti-miR-30b-5p, this suppression was abated, accompanied with restoration of CCL16 (Figure 6a–g). Collectively, silencing of CCL16 migrated LPS-induced apoptosis and inflammation in human lung fibroblast cells *in vitro* relying on miR-30b-5p upregulation.

### 3.6 XIST knockdown downregulated CCL16 expression through sponging miR-30b-5p

**Figure 7:**
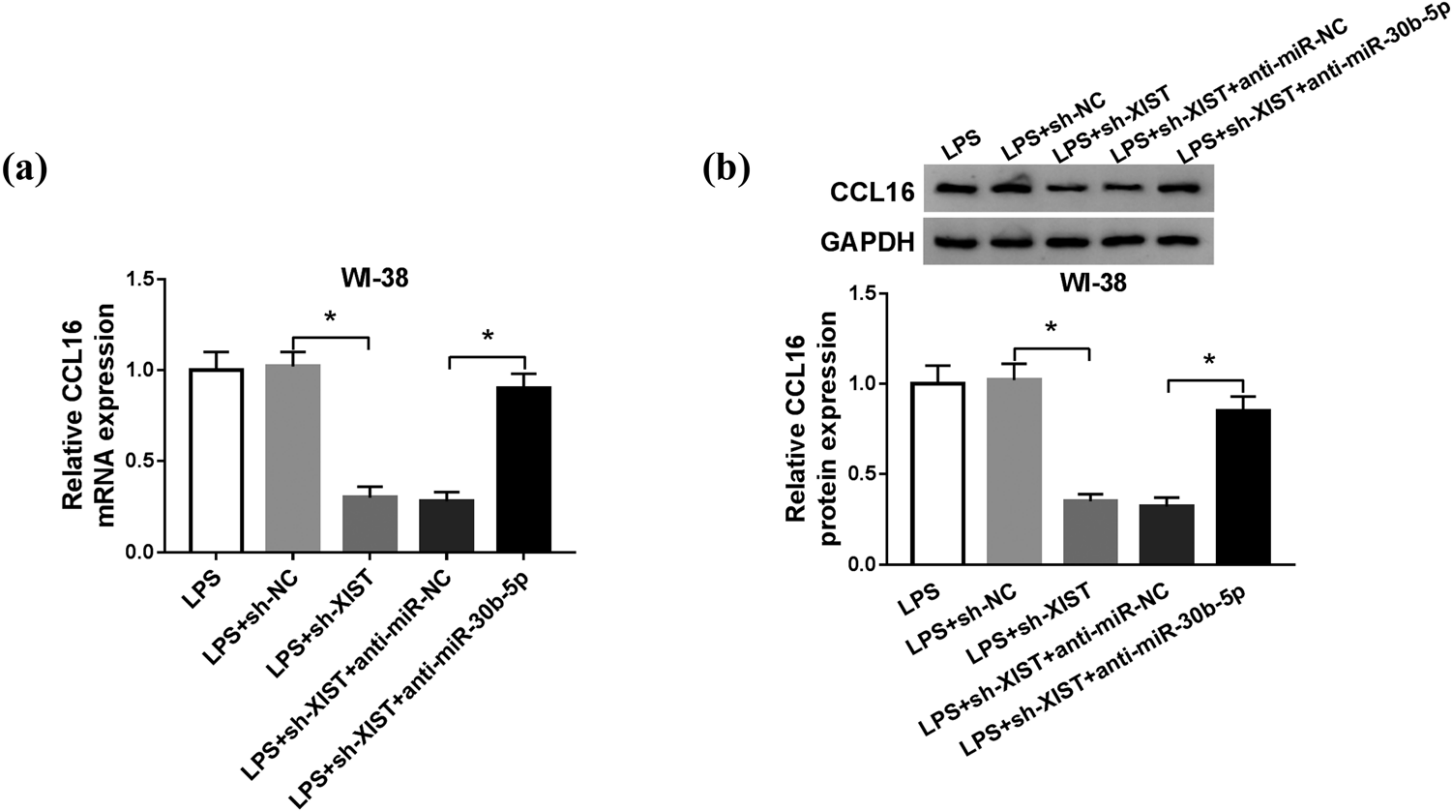
XIST knockdown downregulated CCL16 expression through sponging miR-30b-5p. WI-38 cells were transfected with sh-XIST or sh-NC, co-transfected with sh-XIST and anti-miR-30b-5p or anti-NC, and then treated with 10 μg/mL of LPS for 24 h. (a and b) RT-qPCR and western blotting determined CCL16 expression on mRNA level and protein level. *P < 0.05.

Considering that XIST/miR-30b-5p axis and miR-30b-5p/CCL16 axis had been confirmed, we wondered the reciprocity of XIST and CCL16. WI-38 cells were transfected with sh-XIST or sh-NC, co-transfected with sh-XIST and anti-miR-30b-5p or anti-NC, and then treated with 10 μg/mL of LPS for 24 h. CCL16 expression model was monitored using RT-qPCR and western blotting. As a result, XIST knockdown caused a dramatically low level of CCL16, and simultaneous downregulation of XIST and miR-30b-5p resulted in an elevated level of CCL16 in LPS-induced WI-38 cells (Figure 7a and b). These data showed that XIST could negatively regulate CCL16 expression via miR-30b-5p.

### 3.7 XIST suppressed LPS-activated TLR4/NF-κB signaling pathway by regulating miR-30b-5p/CCL16 axis

**Figure 8:**
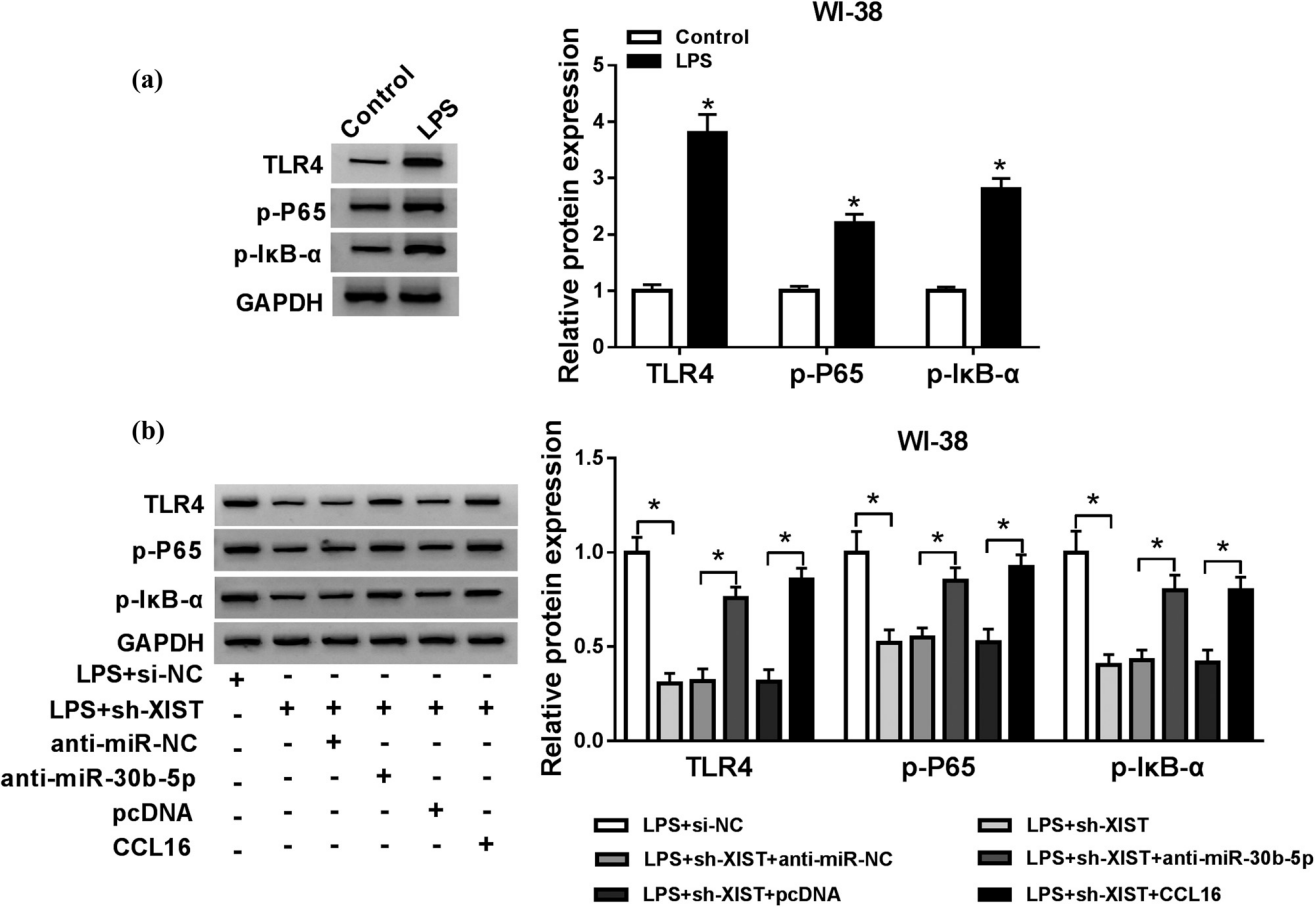
LPS-activated TLR4/NF-κB signaling pathway was regulated by XIST/miR-30b-5p/CCL16 axis. (a and b) Western blotting examined protein expression of TLR4 and p-P65 and p-IκB-α in (a) WI-38 cells treated with 10 μg/mL of LPS for 24 h, and (b) WI-38 cells transfected with si-NC alone, sh-XIST alone or together with anti-miR-NC, anti-miR-30b-5p, and sh-CCL16 alone or together with anti-miR-NC, anti-miR-30b-5p. *P < 0.05.

Western blotting assay revealed that expression of TLR4, p-P65, and p-IκB-α was highly induced in WI-38 cells in response to LPS treatment (Figure 8a); moreover, elevated TLR4, p-P65, and p-IκB-α in LPS-induced WI-38 cells were distinctively attenuated by introducing either sh-XIST or sh-CCL16, which was further overturned by additional introduction of anti-miR-30b-5p (Figure 8b). These data revealed that LPS induced activation of TLR4/NF-κB signaling pathway in WI-38 cells, and XIST knockdown suppressed TLR4/NF-κB activation by regulating miR-30b-5p/CCL16 axis.

## 4 Discussion

Although the connection between lncRNA and pneumonia remains to be uncovered in the upcoming years, several lncRNAs have been suggested in regulating LPS-induced cell models in pneumonia. For example, lncRNA HAGLROS knockdown increased cell viability and decreased apoptosis and autophagy of LPS-induced WI-38 cells by modulating miR-100 [21]. Overexpression of lncRNA TUG1 and SNHG16 reduced cell viability and promoted cell apoptosis and release of inflammatory cytokines in human lung fibroblast cell lines WI-38 and MRC-5 evoked by LPS through inhibiting miR-127 and miR-146a-5p [10,22]. In our work, inspired by these studies, we aimed to detect the role of XIST in LPS-stimulated pneumonia in WI-38 cells, as well as determine a novel XIST/miR-30b-5p axis regulating TLR4/NF-κB signaling pathway.

Here, we observed that XIST expression was significantly upregulated in the serum of patients with pneumonia and LPS-treated WI-38 cells. Functionally, cell viability of WI-38 cells under LPS treatment was declined, and the apoptosis was elevated along with higher IL-6, IL-1β and TNF-α expression and secretion levels. Moreover, these negative influences were considerably restrained with pre-transfection of sh-XIST, which suggested that XIST downregulation might contribute to resistance to LPS-induced inflammatory injury in human lung fibroblast cells *in vitro*. Moreover, these results were in line with the findings of Zhang et al., who discovered a XIST/miR-370-3p/TLR4 pathway in acute pneumonia [15]. Similarly, we indicated a new XIST/miR-30b-5p/CCL16 axis and TLR4/NF-κB signaling pathway. Taken together, both the investigations suggested a protective role of XIST knockdown in LPS-induced inflammatory injury in WI-38 cells via sponging miRNAs, TLR4, and NF-κB pathways, thereby providing a novel target for the treatment of pneumonia. Except for lung fibroblast cells (WI-38 and MRC-5), several other cell types could also be used to establish LPS-induced pneumonia model, such as macrophages and epithelial cells [23,24].

Compared with lncRNA, miRNA is more thoroughly studied. The expression profiles of miRNA were reported in infected patients with pneumonia and LPS-induced mice [25,26]. MiRNA-30b-5p has been revealed to play an important role in lung cancers [27]. Moreover, miR-30b-5p was previously shown to have low expression level in children with pneumonia [19], which was in favor with our data. In that research, protein suppressor of cytokine signaling 3 was proposed as a downstream target of miR-30b-5p in mice macrophages; here, we identified CCL16 to be a novel target gene in human lung fibroblast cells. Similar to the miR-30b-5p expression model in mice induced by LPS, we noticed that it was downregulated in LPS-evoked human lung fibroblast cell line WI-38. However, that research did not further figure out the detailed cellular activities of miR-30b-5p under inflammatory condition. Here, we investigated the effect of miR-30b-5p dysregulation on LPS-stimulated WI-38 cells and found that silencing of this miRNA could partially abrogate the suppression of XIST knockdown on apoptosis and expression of pro-inflammatory cytokines IL-6, IL-1β, and TNF-α. These findings suggested a potential protective role of miR-30b-5p in pneumonia. Moreover, this miRNA was also claimed to take part in LPS-induced kidney injury and liver injury [17,28]. Taken together, miR-30b-5p might be an essential regulator in LPS-induced inflammatory injury among tissues.

CCL16, a liver-expressed chemokine, belongs to human CC chemokines, which play a key role in pneumonia development [24,29,30]. Emerging research studies annotate a pathological role of CCL16 in inflammatory diseases [31,32], implying CCL16 as a powerful inflammatory cytokine. Cappello et al. [33] stated that the effect of CCL16 was as strong as LPS and IFN-γ in macrophages. Therefore, CCL16 was a potent candidate for the target of XIST/miR-30b-5p axis in pneumonia. As a target gene for miR-30b-5p, CCL16 downregulation could alleviate LPS-induced cell injury, as described by improved cell viability, and diminished apoptosis and release of inflammatory cytokines in WI-38 cells. This study supported the findings of Guo et al. [24], which showed that lower expression of CCL16 could not only reverse LPS-induced cell viability inhibition, apoptosis, and overproduction of inflammatory cytokines but also decrease autophagy in human lung epithelial A549 cells. Nonetheless, the autophagy was not investigated in the present study. Besides, the key signaling pathways including NF-κB and TLR4 were discovered to be highly induced in LPS-induced pneumonia in WI-38 cells, and this activation was suppressed by silencing CCL16, which was consistent with previous data [24].

In conclusion, we showed that XIST was upregulated in human pneumonia, and its knockdown could suppress the inflammatory injury in human lung fibroblast cells *in vitro* induced by LPS. The molecular mechanism underlying this protection was through modulating miR-30b-5p/CCL16 axis. This study might enrich the understanding of lncRNA in the occurrence and development of pneumonia and provide a new target for the treatment.

## Appendix

**Table A1:** The raw numerical data from Figure 1

A		B		C	
Health	Pneumonia	Health	Pneumonia	Health	Pneumonia
0.98	3.8	1.72	1.3	3.8	1.3
1.43	4.05	1.73	1.06	4.05	1.06
0.86	3.3	1.91	1.25	3.3	1.25
1.41	4.3	1.91	1.01	4.3	1.01
0.66	4.25	2.51	1.05	4.25	1.05
0.78	3.62	1.86	1.12	3.62	1.12
1.22	5.33	1.94	0.89	5.33	0.89
0.85	3.78	1.81	0.78	3.78	0.78
1.33	3.26	1.83	1.01	3.26	1.01
0.8	3.55	2.03	1.4	3.55	1.4
1.11	4.2	2.12	0.68	4.2	0.68
1.5	3.2	2.45	1.25	3.2	1.25
1.55	3.92	2.09	1.15	3.92	1.15
0.9	3.48	2.91	0.97	3.48	0.97
1.23	3.8	2.67	0.92	3.8	0.92
1.1	4.83	2.53	0.8	4.83	0.8
1.32	4.73	2.45	0.89	4.73	0.89
1.23	4.18	2.48	0.84	4.18	0.84
0.7	4.51	2.45	0.92	4.51	0.92
1.24	4.28	2.65	0.76	4.28	0.76
1.08	4.85	3.22	0.78	4.85	0.78
1.1	2.9	3.21	1.07	2.9	1.07
1.36	3.68	2.74	1.03	3.68	1.03
1.28	3.26	2.79	0.87	3.26	0.87
1.16	3.43	3.16	1.53	3.43	1.53
0.75	3.6	2.43	1.03	3.6	1.03
1.28	3.2	2.34	1.35	3.2	1.35
1.56	3.7	2.85	1.38	3.7	1.38
1.47	3.35	2.36	1.45	3.35	1.45
1.62	3.77	2.49	1.2	3.77	1.2

	0 µg/mL			5 µg/mL			10 µg/mL			20 µg/mL		
D	1.02	1.1	0.88	2.02	2.23	1.82	3.04	3.26	2.86	3.98	4.23	3.65
E	0.98	1.08	0.94	0.71	0.79	0.62	0.52	0.58	0.42	0.33	0.37	0.26

**Table A2:** The raw numerical data from Figure 2

A	0 µg/mL			5 µg/mL			10 µg/mL			20 µg/mL		
	102	110	88	76	83	68	54	61	48	32	36	27
B	0 h			12 h			24 h			48 h		
	102	110	88	76	83	70	52	58	45	43	48	37
C	0 µg/mL			5 µg/mL			10 µg/mL			20 µg/mL		
	6.1	6.8	5.2	12.3	13.8	11	18.2	19.9	16.7	23.8	25.1	21.5
D	0 μg/mL			5 µg/mL			10 μg/mL			20 μg/mL		
Bcl-2	1.02	1.1	0.88	0.75	0.82	0.67	0.51	0.58	0.42	0.31	0.38	0.25
Bax	1.05	1.08	0.87	1.52	1.63	1.42	1.95	2.12	1.78	2.41	2.56	2.3
Cleaved-caspase-3	0.97	1.08	0.95	1.72	1.86	1.57	2.23	2.45	2.02	3.05	3.25	2.82
E	0 μg/mL			5 μg/mL			10 μg/mL			20 μg/mL		
IL-6	52	63	42	408	436	372	709	745	653	912	968	853
IL-1β	32	42	25	305	338	275	545	586	512	709	756	675
TNF-α	65	78	48	607	639	567	805	853	752	1236	1305	1150
F	0 μg/mL			5 μg/mL			10 μg/mL			20 μg/mL		
IL-6	1.05	1.08	0.87	2.03	2.2	1.85	3.52	3.78	3.25	4.53	4.78	4.23
IL-1β	1.01	1.1	0.89	1.85	1.99	1.65	3.05	3.26	2.85	3.8	2.98	3.56
TNF-α	0.99	1.09	0.92	2.75	2.88	2.56	4.05	4.31	3.75	6.05	6.38	5.65

**Table A3:** The raw numerical data from Figure 3

A	LPS			LPS+sh-NC			LPS+sh-XIST					
	1.01	1.1	0.89	1.02	1.1	0.93	0.35	0.42	0.3			
B	Control			LPS			LPS+sh-NC			LPS+sh-XIST		
	98	108	94	52	58	45	47	55	41	85	91	80
C	Control			LPS			LPS+sh-NC			LPS+sh-XIST		
	7.1	7.8	6.2	19.5	20.6	18.5	18.3	19.2	17.3	11.2	12.1	10.3
D	Control			LPS			LPS+sh-NC			LPS+sh-XIST		
Bcl-2	1.02	1.1	0.88	0.51	0.58	0.42	0.45	0.53	0.4	0.85	0.92	0.78
Bax	0.97	1.08	0.95	1.92	2.05	1.81	2.02	2.1	1.91	1.25	1.39	1.12
Cleaved-caspase-3	1	1.08	0.92	2.25	2.38	2.1	2.36	2.48	2.23	1.45	1.58	1.25
E	Control			LPS			LPS+sh-NC			LPS+sh-XIST		
IL-6	48	62	35	723	782	665	705	756	652	253	278	223
IL-1β	30	42	20	602	653	532	581	632	535	182	201	162
TNF-α	65	82	47	853	923	789	875	936	815	356	389	325
F	Control			LPS			LPS+sh-NC			LPS+sh-XIST		
IL-6	1.01	1.1	0.89	3.56	3.75	3.35	3.25	3.45	3.06	1.63	1.78	1.45
IL-1β	1.03	1.09	0.88	3.06	3.26	2.85	3.05	3.18	2.86	1.52	1.69	1.42
TNF-α	0.99	1.07	0.94	4.02	4.25	3.75	4.18	4.37	4.03	2.01	2.15	1.83

**Table A4:** The raw numerical data from Figure 4

B	miR-NC			miR-30b-5p											
WT-XIST	1.05	1.1	0.85	0.38	0.43	0.3									
MUT-XIST	0.96	1.1	0.94	1.02	1.1	0.95									
C	LPS			LPS+sh-NC			LPS+sh-XIST			LPS+pcDNA			LPS+XIST		
	0.98	1.08	0.94	0.98	1.06	0.9	0.35	0.41	0.31	1.02	1.1	0.88	5.02	5.46	4.65
D	LPS			LPS+sh-NC			LPS+sh-XIST			LPS+pcDNA			LPS+XIST		
	1.01	1.08	0.96	0.98	1.05	0.9	1.92	2.06	1.81	1	1.1	0.9	0.38	0.45	0.3
E	LPS			LPS+sh-NC			LPS+sh-XIST			LPS+sh-XIST+anti-miR-NC			LPS+sh-XIST+anti-miR-30b-5p		
	1.01	1.1	0.89	0.98	1.05	0.91	1.85	1.96	1.75	1.92	2.05	1.83	1.25	1.36	1.17
F	LPS			LPS+sh-NC			LPS+sh-XIST			LPS+sh-XIST+anti-miR-NC			LPS+sh-XIST+anti-miR-30b-5p		
	51	53	47	53	58	48	85	89	79	89	92	83	62	67	55
G	LPS			LPS+sh-NC			LPS+sh-XIST			LPS+sh-XIST+anti-miR-NC			LPS+sh-XIST+anti-miR-30b-5p		
	19.2	20.3	18.1	19.3	20.2	18.3	10.2	11.6	9.3	9.5	10.6	8.5	17.3	18.8	16
H	LPS			LPS+sh-NC			LPS+sh-XIST			LPS+sh-XIST+anti-miR-NC			LPS+sh-XIST+anti-miR-30b-5p		
Bcl-2	1.02	1.1	0.88	1.02	1.1	0.88	1.82	1.96	1.75	1.78	1.89	1.62	1.1	1.18	1.03
Bax	1	1.08	0.92	0.98	1.05	0.9	0.45	0.52	0.37	0.47	0.55	0.4	0.92	0.98	0.85
Cleaved-caspase-3	0.97	1.08	0.95	1.02	1.1	0.96	0.33	0.38	0.26	0.31	0.37	0.25	0.87	0.92	0.81
I	LPS			LPS+sh-NC			LPS+sh-XIST			LPS+sh-XIST+anti-miR-NC			LPS+sh-XIST+anti-miR-30b-5p		
IL-6	706	753	658	753	796	715	253	289	221	281	321	254	607	653	554
IL-1β	550	589	515	504	545	471	181	221	145	201	235	175	456	489	421
TNF-α	805	856	751	855	897	801	350	395	315	376	396	342	701	756	643
J	LPS			LPS+sh-NC			LPS+sh-XIST			LPS+sh-XIST+anti-miR-NC			LPS+sh-XIST+anti-miR-30b-5p		
IL-6	1.1	0.9	1	1.02	1.1	0.93	0.45	0.52	0.4	0.44	0.48	0.4	0.85	0.92	0.8
IL-1β	1.02	1.1	0.88	1.03	1.13	0.9	0.4	0.47	0.35	0.4	0.47	0.35	0.9	0.96	0.83
TNF-α	0.95	1.09	0.96	0.97	1.08	0.88	0.35	0.4	0.3	0.33	0.37	0.26	0.92	0.99	0.85

**Table A5:** The raw numerical data from Figure 5

B	miR-NC			miR-30b-5p											
CCL16 3'UTR-WT	1	1.1	0.9	0.38	0.45	0.32									
CCL16 3'UTR-MUT	1.05	1.08	0.87	0.97	1.05	0.9									
D	0 μg/mL			5 μg/mL			10 μg/mL			20 μg/mL					
	1.01	1.1	0.89	2.53	2.69	2.32	3.81	3.96	3.62	4.85	5.01	4.61			
E	0 μg/mL			5 μg/mL			10 μg/mL			20 μg/mL					
	0.98	1.07	0.95	1.72	1.85	1.62	2.41	2.53	2.32	3.15	3.29	3.05			
G	LPS			LPS+miR-NC			LPS+miR-30b-5p			LPS+anti-miR-NC			LPS+anti-miR-30b-5p		
	1.02	1.1	0.88	0.96	1.08	0.96	7.05	7.52	6.62	1.02	1.09	0.89	0.35	0.4	0.3
H	LPS			LPS+miR-NC			LPS+miR-30b-5p			LPS+anti-miR-NC			LPS+anti-miR-30b-5p		
	1.02	1.08	0.9	1	1.1	0.9	0.29	0.36	0.22	0.98	1.06	0.9	2.32	2.41	2.23
I	LPS			LPS+miR-NC			LPS+miR-30b-5p			LPS+anti-miR-NC			LPS+anti-miR-30b-5p		
	1.02	1.1	0.88	0.98	1.07	0.95	0.35	0.41	0.3	1.02	1.1	0.95	1.92	2.01	1.85

C		F	
Health	Pneumonia	miR-30b-5p	CCL16
0.6	2.55	1.3	2.55
0.66	2.38	1.06	2.38
0.68	2.65	1.25	2.65
0.45	3.05	1.01	3.05
0.4	3.28	1.05	3.28
1.03	2.32	1.12	2.32
0.95	2.9	0.89	2.9
0.38	3.15	0.78	3.15
0.54	2.2	1.01	2.2
0.95	2.79	1.4	2.79
0.5	1.96	0.68	1.96
0.54	1.75	1.25	1.75
0.53	1.98	1.15	1.98
0.59	2.68	0.97	2.68
1.06	2.76	0.92	2.76
0.82	3	0.8	3
1.35	3.25	0.89	3.25
1.55	3.2	0.84	3.2
1.28	3.18	0.92	3.18
0.8	3.15	0.76	3.15
0.9	3.1	0.78	3.1
0.9	1.5	1.07	1.5
1	2.38	1.03	2.38
1.1	2.15	0.87	2.15
1	1.89	1.53	1.89
0.42	1.56	1.03	1.56
1.15	1.4	1.35	1.4
0.88	1.45	1.38	1.45
1.2	1.66	1.45	1.66
1.16	1.7	1.2	1.7

**Table A6:** The raw numerical data from Figure 6

A	LPS			LPS+sh-NC			LPS+sh-CCL16			LPS+sh-CCL16+anti-miR-NC			LPS+sh-CCL16+anti-miR-30b-5p		
	1.01	1.1	0.89	1.02	1.1	0.9	0.35	0.4	0.3	0.38	0.45	0.32	0.84	0.89	0.76
B	LPS			LPS+sh-NC			LPS+sh-CCL16			LPS+sh-CCL16+anti-miR-NC			LPS+sh-CCL16+anti-miR-30b-5p		
	0.98	1.08	0.94	0.98	1.06	0.9	0.4	0.46	0.35	0.38	0.43	0.32	0.8	0.87	0.75
C	LPS			LPS+sh-NC			LPS+sh-CCL16			LPS+sh-CCL16+anti-miR-NC			LPS+sh-CCL16+anti-miR-30b-5p		
	51	56	46	48	53	43	89	93	84	86	92	81	65	69	57
D	LPS			LPS+sh-NC			LPS+sh-CCL16			LPS+sh-CCL16+anti-miR-NC			LPS+sh-CCL16+anti-miR-30b-5p		
	19.5	20.6	18.2	19.2	20.3	18.8	10.2	11.1	9.3	9.8	10.7	9.1	17.2	18.1	16.5
E	LPS			LPS+sh-NC			LPS+sh-CCL16			LPS+sh-CCL16+anti-miR-NC			LPS+sh-CCL16+anti-miR-30b-5p		
Bcl-2	1.02	1.1	0.88	1.02	1.08	0.95	2.05	2.12	1.95	2.09	2.16	1.93	1.25	1.39	1.12
Bax	0.98	1.08	0.94	0.98	1.06	0.92	0.4	0.46	0.35	0.38	0.45	0.3	0.85	0.92	0.8
Cleaved-caspase-3	1	1.1	0.9	0.96	1.03	0.89	0.31	0.37	0.25	0.33	0.37	0.27	0.92	0.98	0.85
F	LPS			LPS+sh-NC			LPS+sh-CCL16			LPS+sh-CCL16+anti-miR-NC			LPS+sh-CCL16+anti-miR-30b-5p		
IL-6	705	756	645	725	789	656	263	305	223	281	305	247	652	705	605
IL-1β	625	678	589	602	658	542	195	225	162	205	236	172	500	553	468
TNF-α	872	925	825	851	910	802	401	436	365	420	458	385	701	752	642
G	LPS			LPS+sh-NC			LPS+sh-CCL16			LPS+sh-CCL16+anti-miR-NC			LPS+sh-CCL16+anti-miR-30b-5p		
IL-6	1.02	1.1	0.88	1.02	1.05	0.93	0.38	0.45	0.32	0.36	0.42	0.3	0.88	0.95	0.82
IL-1β	0.95	1.1	0.95	0.97	1.06	0.9	0.43	0.48	0.35	0.45	0.51	0.38	0.92	0.96	0.87
TNF-α	0.98	1.08	0.94	1	1.08	0.92	0.35	0.4	0.3	0.33	0.37	0.29	0.84	0.9	0.78

**Table A7:** The raw numerical data from Figure 7

	LPS			LPS+sh-NC			LPS+sh-XIST			LPS+sh-XIST+anti-miR-NC			LPS+sh-XIST+anti-miR-30b-5p		
A	0.98	1.1	0.92	1.02	1.08	0.96	0.3	0.36	0.25	0.28	0.33	0.22	0.9	0.96	0.83
B	1.02	1.1	0.88	1.02	1.1	0.95	0.35	0.41	0.3	0.32	0.37	0.28	0.85	0.92	0.8

**Table A8:** The raw numerical data from Figure 8

A	Control			LPS		
TLR4	1.02	1.1	0.88	3.82	4.12	3.45
p-P65	1	1.08	0.92	2.2	2.36	2.05
p-IκB-α	0.97	1.08	0.95	2.81	2.99	2.6

B	LPS+si-NC			LPS+sh-XIST			LPS+sh-XIST+anti-miR-NC			LPS+sh-XIST+anti-miR-30b-5p			LPS+sh-XIST+pcDNA			LPS+sh-XIST+CCL16		
TLR4	1	1.08	0.92	0.3	0.36	0.25	0.32	0.38	0.25	0.75	0.82	0.7	0.31	0.38	0.25	0.85	0.92	0.8
p-P65	1.02	1.1	0.88	0.52	0.59	0.45	0.55	0.6	0.5	0.85	0.92	0.78	0.53	0.59	0.45	0.92	0.99	0.86
p-IκB-α	1.05	1.08	0.87	0.4	0.46	0.35	0.43	0.48	0.37	0.8	0.88	0.72	0.42	0.48	0.35	0.8	0.87	0.73
